# Dyslexia with and without Irlen syndrome: A study of influence on abilities and brain‐derived neurotrophic factor level

**DOI:** 10.1002/ibra.12080

**Published:** 2022-12-01

**Authors:** Ehab R. Abdelraouf, Ayman Kilany, Mohamed E. Elhadidy, Hala M. Zeidan, Amal Elsaied, Ola M. Eid, Mostafa M. El‐Saied, Rasha Anwar, Neveen H. Nashaat

**Affiliations:** ^1^ Children with Special Needs Research Department, Medical Research and Clinical Studies Institute National Research Centre Cairo Egypt; ^2^ Learning Disability and Neurorehabilitation Research Field, Medical Research Centre of Excellence National Research Centre Cairo Egypt; ^3^ Pediatric Neurology Research Field, Medical Research Centre of Excellence National Research Centre Cairo Egypt; ^4^ Ophthalmology Research Field, Medical Research Centre of Excellence National Research Centre Cairo Egypt; ^5^ Irlen Egypt Center Cairo Egypt

**Keywords:** BDNF level, cognitive abilities, dyslexia, Irlen syndrome

## Abstract

The presence of comorbid Irlen syndrome (IS) in children with developmental dyslexia (DD) may have an impact on their reading and cognitive abilities. Furthermore, the brain‐derived neurotrophic factor (BDNF) was reported to be expressed in brain areas involved in cognitive and visual processing. The aim of this study was to evaluate some cognitive abilities of a group of dyslexic children with IS and to measure and compare the plasma BDNF level to dyslexic children without IS and neurotypical (NT) children. The participants were 60 children with DD (30 in the DD + IS group; 30 in the DD group) and 30 NT children. The Irlen reading perceptual scale, the Stanford Binet intelligence scale, 4th ed, the dyslexia assessment test, and the Illinois test of psycholinguistic abilities were used. The BDNF level was measured using the enzyme‐linked immunosorbent assay. One‐minute writing and visual closure deficits were more prevalent, while phonemic segmentation deficits were less prevalent in the DD + IS group compared to the DD group. The BDNF level in the DD groups was lower than that in NT children (*p* < 0.001). Some reading and non‐reading tasks were influenced by the presence of a coexisting IS. The reduced BDNF level could play a role in the deficits noticed in the abilities of children with DD.

## Graphical abstract

The aim of this study was to evaluate some cognitive abilities of a group of children manifesting developmental dyslexia with Irlen syndrome (IS) and to measure and compare the plasma brain‐derived neurotrophic factor (BDNF) level to dyslexic children without IS and neurotypicals. The participants were 30 children with dyslexia and IS, 30 children showing dyslexia without IS, and 30 neurotypical children. Various tests and scales were used for assessment of reading, writing, and other cognitive abilities. ELISA was used for measuring the BDNF level. Despite the low prevalence of phonological awareness deficits, the visual burden caused by IS influenced the writing performance in children with dyslexia. The plasma level of BDNF in the children showing dyslexia with and without IS was lower than that in neurotypical children (*p* < 0.001), which could have influenced their learning performance.

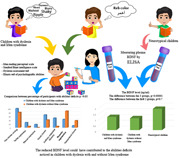

## 1 INTRODUCTION

Irlen syndrome (IS), or Meares–IS, is a visual perceptual disorder that has a negative impact on the reading performance of individuals to variable degrees of severity. Individuals with IS usually show visual and muscular strain during reading. They might show symptoms that include, for example, blurring of printed text, loss of reading place, slow reading, eye strain, narrowing or widening of the eye lids, light sensitivity, watery eyes, sleepiness, headaches, and dizziness.^1^ Some of them may experience difficulties with daily activities such as attention and depth perception.^2^

Developmental dyslexia (DD) is a reading disorder that limits the accuracy and speed of word reading. Learning disorder with impairment in reading is another term for this disorder. About 10% of school children suffer from DD in spite of the absence of intellectual disability and despite receiving proper education. Children with dyslexia demonstrate delays in phonological awareness and auditory processing tasks.^3^ They have been reported to show deficits in motion (moving objects) processing tests and disorders in visuospatial attention.^4^ They show hypoactivation within posterior brain areas, including left‐hemispheric temporoparietal and occipitotemporal regions, which are responsible for auditory and visual information processing.^5^ Moreover, deficits in the magnocellular system and visual working memory have been suggested to be linked to DD and IS.^2^, ^6^, ^7^ Consequently, visual strain attributed to IS could influence reading skills and contribute more burden to children suffering from DD. It has been estimated that about 31%–45% of dyslexic readers show IS in the United Kingdom.^1^, ^8^ Good magnocellular functioning is important for stable binocular fixation and high motion sensitivity. Hence, proper development of orthographic skills depends, amongst others, on proper development of the magnocellular system, which is also involved in photosensitivity.^9^ Moreover, proper development of areas responsible for visual and auditory processing, memory, and attention is mandatory for reading development. Therefore, factors responsible for proper neurological development are suspected to be altered in DD and IS. Brain‐derived neurotrophic factor (BDNF) is one of the substances that are essential for neuronal growth, differentiation, and survival. It regulates synaptic transmission in all brain areas, particularly those associated with learning and memory, such as the cortex, the hippocampus, and the hypothalamus, not to mention its expression in the retina, particularly retinal ganglion cells.^10^, ^11^ All these areas are suspected to play a role in DD and IS pathogenesis. Therefore, the aim of this study was to evaluate the different cognitive, reading, and spelling abilities of a group of children with DD and coexisting IS and to measure the level of plasma BDNF in these children to tackle the possible deficits in their abilities when compared to children with DD without IS and to investigate any change in BDNF levels when compared to children with DD without IS and NT children. This would underline the influence of IS on the performance of dyslexic children and would highlight the possible abilities that could be severely affected in dyslexic children with this syndrome. This will lead to better management of such children and a proper design of intervention plans. Besides, identifying a change in BDNF levels would offer a new marker to be targeted in future studies to determine the cause–effect relationship of these disorders.

## 2 MATERIALS AND METHODS

### 2.1 Study design, participants, and procedures

This was an observational cross‐sectional study that followed the checklist for strengthening the reporting of observational studies in epidemiology (STROBE). A total of 90 participants who were enrolled in the national education system were included in this study. They were native Arabic‐speaking Egyptians of the same socioeconomic status. There were 60 children with DD with poor scholastic achievement. They visited the learning disability and neurorehabilitation research clinic and the pediatric neurology research clinic in the Medical Research Centre of Excellence, National Research Centre, Cairo, Egypt. Inclusion criteria were the diagnosis of DD according to the criteria of the diagnostic and statistical manual of mental disorders‐fifth edition (DSM‐5)^12^ and chronological age range between 8 and 10.5 years. Children with abnormal general, neurological, or ophthalmological examination, uncorrected errors of refraction, other neuropsychiatric comorbidities, suspected syndromic etiology, intellectual disability, obesity, convulsions, and a history of developmental motor delay were excluded. The participants with dyslexia were subjected to clinical interviewing and examination, ophthalmological examination, checking of proper correction for any detected errors of refraction, and electroencephalogram (EEG) evaluation. The Irlen Reading Perceptual Scale was used for group sorting of dyslexic children. The dyslexic participants were divided into two groups according to the presence or absence of coexisting IS: a group that included children with DD + IS (*N* = 30; age: 9.3 ± 1.3; 21 males and 9 females) and a group that included children with DD without IS (*N* = 30; age: 9.1 ± 0.7; 19 males and 11 females). The neurotypical (NT) children were volunteers who agreed to participate in the study (*N* = 30; age: 9.1 ± 0.8; 20 males and 10 females). They were included if their chronological age was between 8 and 10.5 years, and they were performing well in school. Children with a history of developmental delay or any neuropsychiatric disorder or those with 3 or more positive symptoms on the IS self‐test questionnaire^13^ were excluded. Blood samples were obtained from all participants in the morning. The level of BDNF was detected in plasma using the sandwich ELISA method (enzyme‐linked immunosorbent assay) using the human BDNF ELISA kit.^14^ Written informed consents were obtained from the parents of all participants. The study was approved by the Medical Research Ethics Committee of the National Research Centre (approval number: 19237).

### 2.2 Measures

The Irlen Reading Perceptual Scale (IRPS) was performed at the learning disability research clinic of the medical Research Centre of Excellence, National Research Centre, or the Irlen Egypt Center. This scale can be used in individuals starting from the age of 8 years. The IRPS® is divided into four sections. Section 1 provides data on reading difficulties and degree of discomfort. The raw scores of this section were transformed into scaled scores using a formula and were used to determine the presence of IS and to determine the severity of the condition when present using a qualitative scale. Scores 1–3 indicated a mild grade of manifestations, scores 4–7 indicated a moderate grade of manifestations, and scores 8–17 indicated a severe grade of manifestations. These scores were also used for correlation analysis. Section 2 presents observations of Box A, Box B, and Pumpkin tests, which are shapes that are designed to promote visual distortions if present to confirm the statements of Section 1. Section 3 presents the subject's reactions when reading on a white sheet, and the degree of improvement when colored transparencies are used. Section 4 provides information about the types of distortion of written text on reading (such as rivers, swirl, etc).^15^

The Stanford Binet intelligence scale, fourth edition, gives scores for the total IQ together with four subtests, including verbal reasoning, abstract/visual reasoning, quantitative reasoning, and short‐term memory. This test can be administered to individuals ranging in age from 2 to 85 years. The raw scores of the subtests were used to obtain scaled scores. Then, mental age was calculated. The IQ for each subtest and a total IQ score for each child were determined using the following formula: IQ = mental age/chronological age ×100. The normal range of the test scores is from 90 to 109. When a child obtained a score of less than 90, he or she was considered to have a delay in this subtest.^16^, ^17^ These scores were used for correlation analysis.

The dyslexia assessment test has 11 subtests. These include rapid naming, bead threading, 1‐min reading, posture stability, phonemic segmentation, 2‐min spelling, backward digit span, nonsense passage reading, 1‐min writing, verbal fluency, and semantic fluency. This test can be administered in individuals ranging in age from 6.5 to 10.5 years. The raw scores were used to obtain scaled scores for each subtest using standardized tables for certain age groups. The scaled scores are in the form of grades (‐, ‐‐, ‐‐‐, 0, +), where the first three scores represent deficits, while scores 0 and + represent good and superior performance. Furthermore, an at‐risk quotient is finally obtained using a formula based on the number of scaled scores obtained by the child. This quotient increases with worse performance in the subtests.^18^, ^19^ These scaled scores were transformed into a qualitative grading system from 1 to 5. Score 1 represents the lowest level and score five represents the highest level of performance. Scores 1, 2, and 3 represent deficits, while scores 4 and 5 represent good or superior performance. This was determined for the correlation analysis.

The Illinois test of psycholinguistic abilities evaluates some abilities related to the proper development of reading and writing. These abilities include auditory reception; visual reception; auditory association; visual association; verbal expression; manual expression; grammatical closure; visual closure; auditory sequential memory; and visual sequential memory. This test can be administered for individuals ranging in age from 2.33 to 10.25 years. The test was applied to obtain raw scores for each child on each subtest. The raw scores of each subtest were converted into scaled scores according to the child's age using standardized tables. These scaled scores reflect the child's performance in the subtests. The deficits in the subtests were determined when the scaled score in a certain subtest was more than 6 scores below the mean of the sum of the scaled scores of all the 10 subtests. For example, when the sum of scaled scores for a child is 360, the mean of his or her scaled scores is 36, and obtaining a scaled score less than 30 in a certain subtest indicates a deficit in this subtest.^20^, ^21^ The scaled scores were used for correlation analysis.

### 2.3 Statistical analysis

The Statistical Package for Social Sciences version 22 was used to analyze the data. For quantitative data, mean and SD (standard deviation) were used, and for qualitative data, number and percentage were used. Testing for normal distribution of quantitative variables was performed using the Kolmogorov–Smirnov test. Comparison between the first two groups was performed regarding the percentage of participants with deficits using the *χ* ^2^ test. Comparison of the BDNF levels between all groups was performed using either ANOVA or an independent *t*‐test. The Spearman correlation coefficient was used for correlation analysis between the scores of the used tests and the level of BDNF in dyslexic children. *p* was considered significant when less than 0.05.

## 3 RESULTS

### 3.1 Results of the Irlen Reading Perceptual Scale (IRPS)

**Figure 1:**
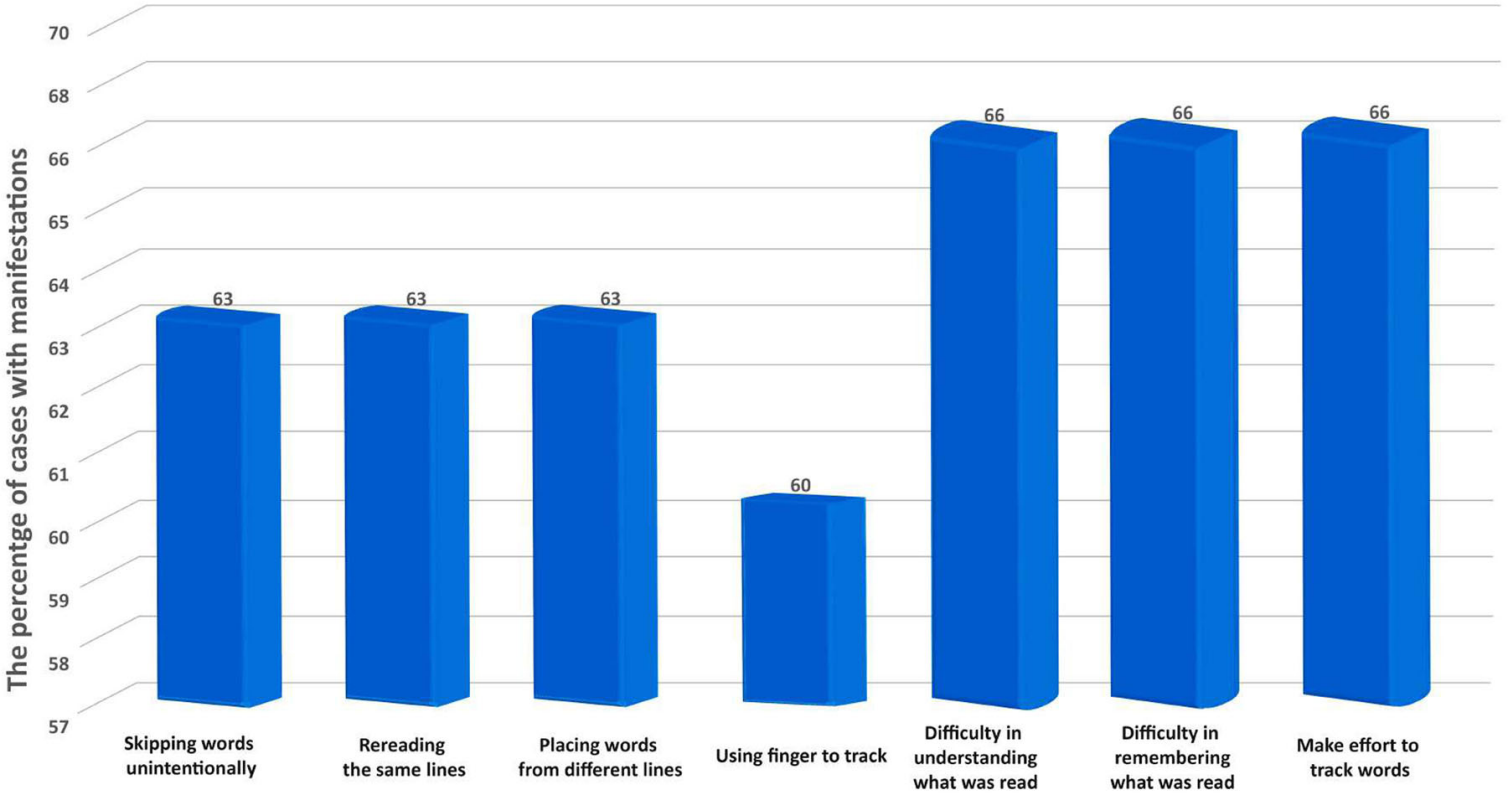
Percentages of cases with some manifestations of reading difficulty in the developmental dyslexia with Irlen syndrome group

In the IRPS, the grade for reading difficulties ranged from 3 to 17. The majority of the participants in the DD + IS group (nearly 86%) showed severe reading difficulties. The discomfort grade ranged from 3 to 14.5. The percentage of participants who showed a moderate grade of discomfort was 60%. The most common reading problems shown by participants were misreading of words and slow reading. The percentage of participants with reading problems exceeded 60% for most of the reading. The percentage of participants who were being bothered by shiny pages was 40% (Table 1, Figure 1). The most common signs of discomfort shown by participants were rubbing of the eyes and eye strain (Table 1).

**Table 1:** Percentages of cases with the most common reading difficulty manifestations and discomfort symptoms in the DD + IS group according to the Irlen reading perceptual scale

Type	Items	Number of participants	Percentage of cases with manifestations
Reading problems	Skipping lines unintentionally	21	70
	Losing place	22	∼73
	Misreading of words	25	∼83
	Avoid reading	21	70
	Slow reading	24	80
	Take frequent breaks	21	70
	Easily distracted	21	70
	Increasing difficulty with prolonged reading	21	70
Discomfort manifestations	Eye strain	20	∼66
	Rubbing eyes	21	70
	Burning eyes	16	∼53
	Feeling sleepy	18	60
	Changing distance from the page	18	60

Abbreviations: DD, developmental dyslexia; IS, Irlen syndrome.

The most common manifestations while doing the tasks in Section 2 are presented in Table 2. All kinds of visual distortions of written texts were reported by the participants, except floating and wavy distortions. The commonly reported distortions were blurriness, star wars, swirl, rivers, and shakiness, while halo, washout, and ripples were less commonly reported among participants in the DD + IS group. The number of visual distortions reported by a single participant ranged from 1 to 7. The majority of participants reported multiple visual distortion patterns. Improvements in symptoms were noticed in the participants after using colored overlays, with considerable improvement in most of the participants. The number of colored overlays used ranged from 1 to 3. The majority of the participants (60%) had colored overlays from the turquoise group.

**Table 2:** The most common manifestations observed in the DD + IS group while performing Box A, Box B, and Pumpkin tests of the Irlen reading perceptual scale

Test	Items	Number of cases with manifestations	Percentage of cases with manifestations
Box A	Distraction while doing the task	19	∼63
	Sense of losing place if you would blink	18	60
Box B	Sense of Difficulty in task	17	∼56
	Distraction while doing the task	18	60
	Losing place	15	50
Pumpkin	Wandering eyes	15	50
	Effort is made to stay at the required place	15	50

Abbreviations: DD, developmental dyslexia; IS, Irlen syndrome.

### 3.2 Results of the other scales and tests used

The IQ of the participants ranged from 85 to 116. The scores of the at‐risk quotients ranged from 1 to 2.8 (mean: 1.629 ± 0.55). The results of the used tests and the percentages of participants with deficits detected among the DD + IS group in the items of the used tests are presented in Tables 3, 4, 5. All participants showed deficits in the 1‐min writing subtest. The next most common deficits among participants of the DD + IS group were rapid naming and nonsense passage reading.

**Table 3:** Results of the Stanford Binet intelligence scale and its subtests and the percentage of cases with deficits in this scale in the DD + IS group

Items	Scores' mean	Standard deviation	Percentage of cases with deficits
Total IQ	96.357	7.09	10
Verbal reasoning	96.286	7.71	16.6
abstract/visual reasoning	98.000	9.75	10
Quantitative reasoning	97.810	10.02	16.6
Short‐term memory	92.524	11.98	23.3

Abbreviations: DD, developmental dyslexia; IS, Irlen syndrome.

**Table 4:** Results of the dyslexia assessment test and the percentage of cases with deficits in its subtests in the DD + IS group

Subtests	Mean of raw scores	Standard deviation	Percentage of cases with deficits
Rapid naming	92.500	37.11	96.6
Bead threading	5.821	1.74	16.6
One‐min reading	15.393	14.50	50
Posture stability	7.357	2.99	23.3
Phonemic segmentation	6.643	3.91	43.3
Two‐min spelling	7.821	6.64	56.6
Backward digit span	3.821	1.67	30
Nonsense passage reading	19.643	15.69	93.3
One‐min writing	6.946	3.10	100
Verbal fluency	3.250	2.45	93.3
Semantic fluency	8.964	3.13	80

Abbreviations: DD, developmental dyslexia; IS, Irlen syndrome.

**Table 5:** Results of the Illinois test of psycholinguistic abilities and the percentage of cases with deficits in its subtests in the DD + IS group

Subtests	Mean of scaled scores	Standard deviation	Percentage of cases with deficits
Auditory reception	27.368	10.14	66.6
Visual reception	32.789	7.56	30
Auditory association	32.052	8.07	30
Visual association	34.578	5.94	20
Verbal expression	31.210	6.04	26.6
Manual expression	34.894	3.11	16.6
Grammatical closure	42.315	6.32	10
Visual closure	27.894	4.38	66.6
Auditory sequential Memory	31	5.54	50
Visual sequential memory	37.052	7.87	10

Abbreviations: DD, developmental dyslexia; IS, Irlen syndrome.

The DD + IS group had higher percentages of participants with deficits in abilities than the DD group, with significant statistical differences in 1‐min writing, verbal expression, manual expression, visual closure, and auditory sequential memory, while phonemic segmentation and grammatical closure were observed less in the DD + IS group (Tables 6, 7, 8).

**Table 6:** Comparison between the DD + IS and the DD groups regarding the percentages of cases with deficits in the Stanford Binet intelligence scale scores

Items	Percentage of cases with deficits in the DD + IS group	Percentage of cases with deficits in the DD group	*p*‐Value
Total IQ	10	6.6	0.2
Verbal reasoning	16.6	6.6	0.2
abstract/visual reasoning	10	20	0.2
Quantitative reasoning	16.6	3.3	0.08
Short‐term memory	23.3	16.6	0.5

Abbreviations: DD, developmental dyslexia; IS, Irlen syndrome.

**Table 7:** Comparison between the DD + IS and the DD groups in terms of the percentages of cases with deficits in the dyslexia assessment test subtests

Subtests	Percentage of cases with deficits in the DD + IS group	Percentage of cases with deficits in the DD group	*p*‐Value
Rapid naming	96.6	100	0.3
Bead threading	16.6	30	0.2
One‐min reading	50	66.6	0.1
Posture stability	23.3	23.3	1
Phonemic segmentation	43.3	86.6	<0.001*
Two‐min spelling	56.6	73.3	0.1
Backward digit span	30	53.3	0.06
Nonsense passage reading	93.3	96.6	0.5
One‐min writing	100	80	0.009*
Verbal fluency	93.3	96	0.5
Semantic fluency	80	64	0.1

Abbreviations: DD, developmental dyslexia; IS, Irlen syndrome.

* Significant statistical difference.

**Table 8:** Comparison between the DD + IS and the DD groups regarding the percentages of cases with deficits in the subsets of the Illinois test of psycholinguistic abilities

Subtests	Percentage of cases with deficits in the DD + IS group	Percentage of cases with deficits in the DD group	*p*‐Value
Auditory reception	66.6	48	0.1
Visual reception	30	20	0.3
Auditory association	30	16	0.2
Visual association	20	15	0.7
Verbal expression	26.6	6.6	0.03*
Manual expression	16.6	0	0.01*
Grammatical closure	10	36.6	0.01*
Visual closure	66.6	40	0.03*
Auditory sequential Memory	50	6.6	<0.001*
Visual sequential memory	10	6.6	0.6

* Abbreviations: DD, developmental dyslexia; IS, Irlen syndrome.

* Significant statistical difference.

### 3.3 Results of BDNF level measurement and its correlation with the scales and tests used

The difference between the DD + IS group and the NT children in the BDNF level was significant, being lower in the DD + IS group. The difference between the DD group and the NT children was significant, being lower in the DD group. The difference between the DD + IS and the DD groups was nonsignificant, being lower in the DD group (Table 9). Correlation analysis between the BDNF level and the scores of the tests used did not reveal any significant statistical difference in both groups.

**Table 9:** Comparison between participants in the DD + IS and the DD groups and neurotypical children regarding the BDNF level

	DD + IS Group	DD group	Neurotypical children	*p*
BDNF level (ng/ml)	1.15 ± 0.35	1.043 ± 0.36	1.86 ± 0.43	<0.00001*, a
BDNF level (ng/ml)	1.15 ± 0.35	1.043 ± 0.36	‐	0.7^b^
BDNF level (ng/ml)	1.15 ± 0.35	‐	1.86 ± 0.43	<0.001*, b
BDNF level (ng/ml)	‐	1.043 ± 0.36	1.86 ± 0.43	<0.001*, b

Abbreviations: BDNF, brain‐derived neurotrophic factor; DD, developmental dyslexia; IS, Irlen syndrome; ml, milliliter; ng, nanogram.

* Significant statistical difference.

^a^ Analysis of variance.

^b^ Independent *t*‐test.

## 4 DISCUSSION

Children with DD have reading and spelling difficulties, which influence their academic performance. The academic difficulties that these children experience have a huge negative impact on their healthy psychological development and on the mental health of their families, not to mention the financial burden of the rehabilitation process. The presence of comorbid IS adds to the challenges faced by such children and increases the effort required to acquire the reading, writing, and spelling tasks. Therefore, identifying the abilities that could be delayed in such children and the impact of visual stress manifestations on the performance of dyslexic children would help in their thorough rehabilitation and would help in better tailoring of proper intervention plans to strengthen their abilities besides the use of colored overlays or glasses. Furthermore, identifying possible biochemical changes in BDNF that is involved in the brain development of children with or without IS will help provide a better understanding of the pathophysiological changes of such complicated disorders.

The majority of the participants in the DD + IS group in this study reported feeling sleepy while reading in fluorescent light and with white background papers against black printed words. Our circadian system is biologically anchored and regulated by short versus long wavelengths associated with day light (e.g., blue light at midday and red light near sunset). It has been found that blue and red lights induce alertness. The blue light induces alertness towards more precise tasks while the red induce alertness towards tasks requiring less precision.^22^, ^23^ The retinal ganglion cells that contain melanopsin are tuned toward short‐wavelength blue light. They are directly connected to subcortical circuits and, therefore, regulate seasonal changes in affect. These cells are connected to the brain via the hypothalamic suprachiasmatic nucleus.^24^ The acute effect of light on melatonin suppression is strongly blue‐shifted, regulating autonomic nervous system activity as well as influencing alertness, thermoregulation, and heart rate. Blue light has been reported to increase the release of ATP in the optic lens and reduce intraocular pressure, which highlights the influence of light on the eye and the optic pathway. Fluorescent light, which is usually used for indoor lighting, has a wavelength between 480 and about 570 nanometers. It is shifted towards the blue light and could cause depletion of ATP, leading to fatigue of neural pathways or dysregulation of intraocular pressure.^22^ This could explain why the visual and muscular strainmanifestations of IS are severe in day light and whileusing indoor fluorescent light in the participants.

Considering that light influences the brain via retinal ganglion cells and the hypothalamic suprachiasmatic nucleus in which BDNF is richly expressed, the reduced BDNF level detected in this study could have had an impact on the functioning of this system and could have contributed to the development of IS stress symptoms and the reduced scores in reading and non‐reading tasks. BDNF can cross the blood–brain barrier.^25^ Therefore, its plasma level could reflect its level in the brain. BDNF deficiency has been linked to abnormal functioning of various brain areas, including cortical regions involved in language processing and language‐based learning, as well as subcortical regions such as the hypothalamus, which is responsible for emotions, behavior, homeostasis, thermoregulation, energy maintenance, endocrinal control, and memory processes.^26^ BDNF is also widely expressed in the hippocampus, which is responsible for memory and learning.^11^ The reduction in its level could be related to the deficits in memory and other abilities noticed in dyslexic participants. The absence of statistically significant correlations between BDNF level and the scores of the tests used could be explained by the complicated role of BDNF and its role in controlling other systems involved in brain functions. BDNF has an influence on other neurotransmitter systems such as serotonin, GABA, glutamate, and dopamine, which are all involved in proper functioning of cognitive brain functions.^27^

Rapid naming, verbal fluency, and semantic fluency deficits were common among participant in both groups. The percentage of participants with deficits was higher in the DD + IS group in terms of verbal and manual expression. These abilities are related to memory performance and higher‐order semantic language abilities, indicating the presence of vocabulary deficits. These findings are consistent with previous reports such as those of Chouinard et al.,^28^ who found that individuals with IS showed deficits in language processing, lexical processing, word retrieval, movement initiation and learning, interlimb coordination, motor imagery, memory, and visuospatial and visuomotor attention. These functions were related to activation differences in individuals with IS when compared to controls in Brodmann area 6 (supplementary motor area) as detected by functional MRI.^28^

It is noteworthy that the percentages of participants with reading ability deficits in both groups were close, and yet, the percentage of participants having phonemic segmentation deficits in the DD group was much higher than that in the DD + IS group. Phonological awareness deficits were strongly linked to DD.^29^ The common deficits in reading and writing abilities, despite the lower percentage of participants with phonological awareness deficits in the DD + IS group, could be attributed to the visual stress manifestations caused by IS. All participants with IS showed writing (copying text) deficits despite not having ophthalmological abnormalities when examined by the ophthalmologist. This could be related either to decoding and reading disorders, which limit the speed of copying a text, or to visual closure deficits and visual distortions of the printed text. Besides, the child's ability to recognize small targets when examined by ophthalmologists is not the only predictor of the ability to see words in which the letters are crowded and clustered.^30^ These visual distortions detected in the participants were in the form of rivers, shakiness, blurriness, washout, swirl, ripple, star wars, or seesaw, either individually or combined.

It has been observed that not only are visual deficits common among IS participants but also auditory, language, and memory abilities. This is in agreement with Garcia et al.,^31^ who reported common associations between auditory and visual processing deficits in school‐aged children with IS. Furthermore, this study highlighted the differences in the abilities between dyslexic children with and without IS. Therefore, a proper and thorough evaluation of the different abilities of children with IS is essential for the optimum tailored management of such children and adequate rehabilitation, in addition to the use of colored overlays or lenses.

It is difficult to judge whether the change in the BDNF level (reduction when compared to NT children) is a cause or a result of the reading disorder and the altered light signal processing, which could be considered a limitation of the study. However, this is the first study that has investigated such measures in children with IS and coexisting dyslexia and drew attention to the presence of changes in biochemical and cognitive abilities in such children. Furthermore, the exclusion criteria were selected to ensure exclusion of participants with disorders that could have an influence on the BDNF level, such as obesity or other neuropsychiatric disorders. Individuals with obesity, attention deficit hyperactivity disorder, and autism spectrum disorder, for example, have been reported to have altered levels of BDNF compared to neurotypical ones.^32^, ^33^, ^34^ Moreover, the age range of the participants was chosen to fit the age range of all the scales and tests used. The tests used in this study enabled the assessment of various cognitive abilities including verbal, nonverbal, fine motor, eye–motor coordination, auditory, visual, and memory abilities, in addition to reading, spelling, and writing performance in dyslexic children with and without IS.^18^, ^20^ This was performed to provide an overview of the cognitive abilities of children with DD who are known to have normal ranges of intelligence quotients.

Conducting future observational prospective cohort study targeting similar younger groups would be beneficial in identifying the cause–effect relationship. Study of the influence of therapy via intervention sessions, medications that help elevate the BDNF level, and prolonged use of colored overlays or lenses on the BDNF level is recommended. The percentage of participants with reading deficits was almost similar in the DD + IS and the DD groups. The BDNF level did not show a significant statistical difference between these two groups. Thus, the presence of dyslexia itself could be related to such a change in the BDNF level. Consequently, measurement of the BDNF level in individuals with IS without dyslexia is recommended in future studies.

## 5 CONCLUSIONS

Deficits in writing, visual closure, verbal and manual expression, and auditory sequential memory abilities were more prevalent among children with dyslexia and IS, while deficits in phonemic segmentation and grammatical closure abilities were less prevalent compared to children with dyslexia without IS. The reduced BDNF level could have contributed to the deficits in abilities noticed in children with dyslexia with and without IS.

## AUTHOR CONTRIBUTIONS

All authors contributed to the study's concept, design, and work, as well as the data analysis and interpretation. All authors edited, reviewed, and approved the final draft of the manuscript.

## CONFLICT OF INTEREST

The authors declare no conflict of interest.

## ETHICS STATEMENT

This study was approved by the medical research ethics committee of the National Research Centre, and was carried out according to the latest version of the Helsinki Declaration of 1975. All parents of the participants signed informed consents.

## TRANSPARENCY STATEMENT

The author confirms that this manuscript is an honest, exact, and transparent description of the reported research. There is no omission or concealment of any important aspect in the study or any differences from the planned study before the start.

## Data Availability

The data sets used and/or analyzed during the current study are available from the corresponding author upon reasonable request.
